# No Association between SARS-CoV-2 Infection and the Polymorphism of the Toll-like Receptor 7 (*TLR7*) Gene in Female Population

**DOI:** 10.3390/diagnostics13233510

**Published:** 2023-11-23

**Authors:** Mohammed Zayed, Yong-Chan Kim, Chang-Seop Lee, Byung-Hoon Jeong

**Affiliations:** 1 Korea Zoonosis Research Institute, Jeonbuk National University, Iksan 54531, Republic of Korea; mzayed2@vet.svu.edu.eg; 2 Department of Bioactive Material Sciences, Institute for Molecular Biology and Genetics, Jeonbuk National University, Jeonju 54896, Republic of Korea; 3 Department of Surgery, College of Veterinary Medicine, South Valley University, Qena 83523, Egypt; 4 Department of Biological Sciences, Andong National University, Andong 36729, Republic of Korea; kych@anu.ac.kr; 5 Department of Internal Medicine, Research Institute of Clinical Medicine, Jeonbuk National University, Jeonju 54907, Republic of Korea; 6 Biomedical Research Institute, Jeonbuk National University Hospital, Jeonju 54907, Republic of Korea

**Keywords:** coronavirus disease 2019, SARS-CoV-2, *TLR7*, genetic variation, female

## Abstract

Coronavirus disease 2019 (COVID-19) is caused by severe acute respiratory syndrome coronavirus 2 (SARS-CoV-2), a single-stranded RNA virus. Toll-like receptor 7 (*TLR7*) recognizes single-stranded RNA viruses. The *TLR7* gene plays a critical role in the human innate and adaptive immune response to SARS-CoV-2 infections. Genetic factors probably affect SARS-CoV-2 infection susceptibility. In the current study, our aim was to search for genetic variations associated with COVID-19 patients in the *TLR7* gene of a Korean population. We designed five gene-specific primers to cover the coding region of the human *TLR7* gene. Using amplicon sequencing, we screened the genetic polymorphisms in the coding region of the *TLR7* gene in COVID-19 patients and healthy controls. The genotype frequencies, allele frequencies, and Hardy–Weinberg equilibrium (HWE) were examined. We identified a low-frequency synonymous single nucleotide polymorphism (SNP) (rs864058) in the coding region of the *TLR7* gene. There were no significant differences in the genotype or allele frequencies of the *TLR7* rs864058 polymorphism between COVID-19 female patients and healthy controls (*p* = 1.0). In conclusion, *TLR7* (rs864058) polymorphism is low frequency in Korean populations and is not associated with SARS-CoV-2 infection.

## 1. Introduction

Coronavirus disease 2019 (COVID-19) is caused by severe acute respiratory syndrome coronavirus-2 (SARS-CoV-2) [1]. SARS-CoV-2 is a positive-sense single-stranded RNA virus with a wide range of hosts, including bats, snakes, pangolins, humans, cats, and dogs [2]. Among clinical symptoms, the most prevalent symptom is fever, followed by cough, myalgia, headache, and sore throat [3]. Patients who required intensive care were older and more likely to have underlying disorders [4]. Tiecco et al. recently reported the stealthy resurgence of COVID-19 as a result of the dissemination of the SARS-CoV-2 variant of concern, Omicron [5]. The authors emphasized the BA.2 lineage and reviewed the virological properties, such as transmissibility, pathogenicity, and resistance to vaccine- and infection-induced immunity, as well as antiviral medicines, raising a public health concern. Various data have shown that the fundamental cause of COVID-19 is an unregulated host immune response, which can potentially lead to a lethal cytokine storm [6,7]. Toll-like receptors (TLRs) play a role in immediate pathogen detection and subsequent activation of innate immunity by stimulating inflammatory responses to eliminate the invading organisms [8,9]. Genetic diversity in TLR genes influences cellular immune response and disease risk [10]. As TLRs have a role in the induction of cytokine storms, they might also be vitally involved in the cytokine storm associated with SARS-CoV-2 infection [11,12]. Consequently, the immune response to SARS-CoV-2 infection and the role of the TLRs are being considered to identify therapeutic approaches [13].

Genetic variation in TLR-encoding genes has been linked to COVID-19’s significant respiratory symptoms [14,15]. Several studies have discovered different TLR genes related to COVID-19 susceptibility through genome-wide association studies or specific gene analysis [16,17]. The following polymorphisms are associated with the prognosis and susceptibility to COVID-19 infection: *TLR3* rs3775290, *TLR4* Asp299Gly, Thr399Ile, and *TLR9* [16]. The *TLR7* gene is expressed in monocytes and dendritic cells that are involved in inflammation and infection, leading to mediating the production of type I interferon (IFN) and other inflammatory cytokines upon stimulation [18]. Van der Made et al. detected loss-of-function variants of the X-chromosomal *TLR7* gene on whole-exome sequencing of four male patients with severe COVID-19 infection [19]. The presence of these rare variants in young men with severe COVID-19 has been studied, resulting in *TLR7* missense variants in 14.3% of the patients [20].

While there is no difference in COVID-19 prevalence between men and women, male patients tend to have more clinical symptoms and a higher risk of requiring intensive care than female patients [21]. It has been suggested that X-linked genes may explain this phenomenon. The 10 *TLR* genes are found on chromosomes 3, 4, 9, and X in a total of 6 chromosome regions. The *TLR7* gene is located on the X chromosome; therefore, studies suggest that SARS-CoV-2 may exhibit a gender-dependent response [22]. The immune cells of females exhibit biallelic *TLR7* expression, producing more inflammatory factors upon *TLR7* stimulation compared to males. This enhanced inflammatory response contributes to low COVID-19 mortality observed in the female population. However, it is important to note that certain factors associated with females, including obesity, changes in menstrual and sleep cycles, and maternal outcomes, are identified as risk factors for COVID-19 mortality [23,24,25]. Genetic variation may explain differences in cytokine production within COVID-19 patients. Single nucleotide polymorphisms (SNPs) in the *TLR7* gene may be associated with *TLR7* gene expression. Given the role that *TLR7* plays in COVID-19, genetic screening of *TLR7* gene polymorphisms in COVID-19 patients must be investigated. 

In the current study, we searched for polymorphisms in the coding region of the *TLR7* gene in the female Korean population using amplicon sequencing. We aimed to identify any link between *TLR7* polymorphisms and their potential influence on COVID-19 susceptibility.

## 2. Materials and Methods

### 2.1. Selection of Participants

This study included 90 healthy females in the control group and 87 patients diagnosed with COVID-19 (Table 1). 

The two groups had similar ages and sexes. COVID-19 patients with a median age of 55.0 years were admitted to Jeonbuk National University Hospital, Jeonju-si, Republic of Korea, between April 2020 and September 2021. Healthy controls with a median age of 61.0 years were unrelated subjects recruited from the Korea Biobank Network. 

### 2.2. Inclusion Criteria

This study included Korean female patients diagnosed with SARS-CoV-2 through polymerase chain reaction (PCR). The patients were categorized into two groups based on clinical symptoms: mild and severe. The mild COVID-19 group included 74 cases with symptoms such as sore throat, arthralgia, and anosmia. The severe COVID-19 group included 13 cases with conditions such as respiratory distress, mechanical ventilation, and low oxygen concentration in arterial blood. A total of 23 patients received vaccinations, including 10 AstraZeneca, 1 Janssen, and 12 Pfizer (6 one dose and 6 two doses).

### 2.3. Exclusion Criteria 

Patients with known HIV, hepatitis B or C, and/or chronic lung diseases were excluded from this study. Additionally, children and pregnant females were also excluded from this research.

### 2.4. Calculation of Sample Size

Using the prevalence rate of COVID-19 in the Korean population [26], the sample size was estimated using the QUANTO program version 1.2.4. The minimum required sample size indicated that 79 subjects were needed for each group with a power of 80% and a significance level of 5%. To enhance statistical analysis, we added an additional 8–20 individuals in each group.

### 2.5. DNA Isolation and Genotyping 

Blood samples (200 μL) were used for the preparation of genomic DNA using a blood genomic DNA isolation kit (Qiagen, CA, USA) following the manufacturer’s directions. To cover the coding region of the *TLR7* gene, five primers were designed for PCR (Table 2). These primers were used to amplify the entire protein-coding region of the human *TLR7* gene (Gene ID: 51284).

The PCR mixture included 1 µL of genomic DNA, 10 pmol of each primer, 2.5 µL of 10 *Taq* DNA polymerase buffer, 0.5 µL of a 0.2 µM dNTP combination, 5 µL of 5 Band Helper, and 0.25 µL *Taq* DNA polymerase (BioFACT, Daejeon, Korea). The PCR conditions were set according to the manufacturer’s instructions. The *TLR7* gene primers were annealed at 58 °C using the C1000 Touch Thermal Cycler (Bio-Rad, Hercules, CA, USA). The PCR findings were visualized using electrophoresis on 1% agarose gel.

The PCR products were purified with the FavorPrep GEL/PCR Purification Kit (Favogen Biotech, Ping Tung, Taiwan) and sequenced using an ABI 3730 sequencer (ABI, Foster City, CA, USA). The sequencing reaction was performed using Applied Biosystems’ BigDye^TM^ Terminator v3.1 kit (Applied Biosystems, Foster City, CA, USA) following the manufacturer’s instructions. The 10 µL sequencing reaction comprised 7.0 µL BigDye™ Terminator v3.1 Ready Reaction Mix, 10 pmol primer, and 50 ng PCR product. The sequencing results were analyzed using Sequencing Analysis Software version 5.3.1 (Applied Biosystems, USA). Finch TV software 1.4.0 (Geospiza Inc., Seattle, WA, USA) was used to visualize sequencing.

### 2.6. Statistical Analysis

The genotype and allele frequencies of the *TLR7* gene were analyzed and compared between the COVID-19 patients and healthy controls by Fisher’s exact test using SAS 9.4 software. Analysis of the Hardy–Weinberg equilibrium (HWE) test was also performed. The age between the two groups was analyzed using median test. Statistical significance was defined as *p* < 0.05, and all *p*-values were two-tailed. 

## 3. Results

There were no statistically significant differences in terms of age between the patients and control groups (*p* = 0.11). 

The sequenced PCR products were found to be identical to the Homo sapiens *TLR7* gene, which was registered in GenBank (Gene ID: 51284).

**Figure 1:**
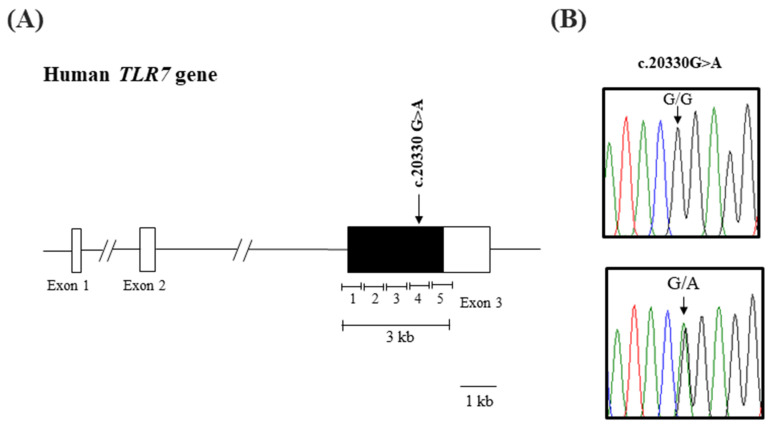
Identification of genetic polymorphisms of the human *TLR7* gene in healthy and COVID-19 patients. (**A**) Simplified the sequenced region map of the human *TLR7* gene. The edged horizontal bar indicates the regions sequenced (3387 bp). Vertical lines indicate the genetic polymorphism identified in this study. (**B**) Electropherograms of a rare single nucleotide polymorphism (SNP) of the *TLR7* gene found in the current study. The colors of the peaks indicate each base of nucleotides (green: adenine; red: thymine; blue: cytosine; and black: guanine).

In our study, we utilized PCR product sequencing data to conduct genotyping of the *TLR7* gene. Analysis of the sequence variation in the coding region of the *TLR7* gene identified a low-frequency and a rare synonymous SNP, c.20330 G>A (rs864058), which does not result in an amino acid replacement, in both healthy controls and patients. The identified SNP is classified as a low-frequency SNP based on its frequency, one frequency in both the healthy controls and patients. The electropherograms displaying the SNP are presented in Figure 1. 

The genotype and allele frequencies of the *TLR7* polymorphisms were assessed through amplicon sequencing of healthy controls and patients, using each primer. The distributions of genotype frequencies, allele frequencies, and HWE for *TLR7* rs864058 in the present study are comprehensively presented in Table 3. 

There was no significant association between *TLR7* rs864058 SNP and SARS-CoV-2 infection.

## 4. Discussion

To investigate the impact of beneficial gene expression of the second X chromosome in females, we recently investigated the potential association between four identified potentially functional SNPs in the promoter region and exon 1 of the *TLR8* gene and COVID-19 susceptibility between healthy control and COVID-19 patient groups in a Korean population. The results, however, revealed no significant difference in the genotype and allele frequencies in the studied population. Therefore, an X chromosomal gene of interest is *TLR7*, which has been identified to be involved in type 1 interferon production in COVID-19 [27,28] needs to be investigated. In the current study, we aimed to search for genetic variations associated with COVID-19 patients in the coding region of *TLR7* gene of a Korean female population. We detected *TLR7* rs864058 SNP, not resulting in an amino acid replacement (rs864058), indicating that females can do better in SARS-CoV-2 infection [21]. The *TLR7* rs864058 genotype frequencies are very low (less than 5%) and did not show any significant differences between COVID-19 patients and healthy controls. It is commonly observed that associations with low-frequency and rare variants have minor impacts on disease [29]. Moreover, the assessment of low-frequency variants often necessitates additional genomic tools, such as genotype imputation and the use of whole-exome or whole-genome sequencing [29].

The genetic background can impact the incidence and consequences of infectious diseases, including H1N1 influenza virus and COVID-19 [30,31,32,33,34,35]. In SARS-CoV-2 infection, TLRs (2–9) play significant roles in detecting the viral particles and stimulating the innate immune system to eliminate the infection [16]. However, *TLR7* is thought to be the most significant among the TLRs that have demonstrated a response to coronaviruses. While *TLR7* expression has been implicated in respiratory syncytial virus-induced lung inflammation [36], several studies have reported associations between COVID-19 and *TLR7* variants. For instance, *TLR7* rs179008 genotypes are associated with an extremely high risk of COVID-19 pneumonia but not with disease outcome [37]. The same study reported that patients with ‘T/T’ genotype of *TLR7* had 4.76 times higher odds of displaying COVID-19 pneumonia compared to patients with the wild homozygous ‘A/A’ genotype. The *TLR7* rs179008 genotype has also been linked to low expression levels of the *TLR7* gene. Another study showed that the *TLR7* rs3853839 GG genotype was considerably more prevalent in COVID-19 patients (38.7%) than in the control individuals (4.4%) [38]. In contrast, the genotype CC was significantly higher amongst controls (56.3%) than cases (24.7%). Thus, the G allele was significantly more prevalent among cases (57.0%), and the C allele was significantly more prevalent among controls [38].

TLRs variants have also been linked to respiratory disorders [39]. The *TLR7* rs179008 polymorphism, for example, is strongly associated with the pathogenesis of bronchial asthma [40]. There is confirmation that polymorphisms in the *TLR7* gene are associated with susceptibility to respiratory viral infections [41]. Zhang et al. showed that *TLR7* and *TLR8* polymorphisms may play an essential role in the pathogenesis of asthma [42]. Several studies have investigated the *TLR7* rs864058 polymorphism in various respiratory diseases, such as allergic rhinitis [43], measles infection [44], and prostate cancer [45]. In SARS-CoV-2 infection, no association studies of *TLR7* rs864058 with COVID-19 susceptibility have been reported. In the current study, we did not find a significant difference in the genotype and allele frequencies of *TLR7* rs864058 between COVID-19 patients and healthy controls in Korean population (*p* > 0.05).

This is one of the few studies investigating the genetic variation associated with SARS-CoV-2 infection among females. The study only investigated the Korean population. Further studies are needed to examine the genetic variation in the *TLR7* gene in other ethnic groups. Additional studies should also investigate other polymorphisms of the *TLR7* gene and their haplotype effects on susceptibility to SARS-CoV-2 infection.

Despite the small sample size in our current investigation, it is noteworthy that analogous studies have previously performed association analyses in similarly small sample sizes to gain initial insights before undertaking extensive validation endeavors in larger populations [46,47,48]. In line with these previous reports, our study conducted a preliminary analysis within a limited cohort, revealing no association between *TLR7* (rs864058) polymorphism and susceptibility to COVID-19. Nevertheless, it is necessary to conclusively affirm the absence of an association between *TLR7* (rs864058) polymorphism and susceptibility to COVID-19 in a larger population using more robust statistical power.

## 5. Conclusions

To our knowledge, this is one of the few studies that have screened genetic variations in the *TLR7* gene in females. According to the results, the *TLR7* (rs864058) polymorphism is low frequency in Korean populations and not associated with SARS-CoV-2 infection.

## Figures and Tables

**Table 1:** Detailed information on the study population.

Characteristics	Healthy Controls	COVID-19 Patients	*p*-Value
Number, *n*	90	87	
Age, median (IQR)	61.0 (49.2–71.7)	55.0 (45–68)	0.11

**Table 2:** Primers used to cover the coding region of the human *TLR7* gene.

Primer Name	Forward	Reverse	Amp. Size	Ann. Temp.
CDS 1	GGTTGGGGATGCTGTTTAGA	GTAGGGACGGCTGTGACATT	806 bp	58 °C
CDS 2	TCTACCTGGGCCAAAACTGT	CAGGACCTGGGGTTCATAACT	851 bp	58 °C
CDS 3	TGAAGTTGGCTTCTGCTCAA	CAGTGGTCAGTTGGTTGTGG	821 bp	58 °C
CDS 4	CCTGGAAACTTTGGACCTCA	GCTGTATGCTCTGGGAAAGG	746 bp	58 °C
CDS 5	GGCCAAGATAAAGGGGTATCA	CAAAACACGCTTTTGGTGTG	619 bp	58 °C

Coding sequence (CDS).

**Table 3:** Comparison of genotype and allele frequencies of the *TLR7* low-frequency polymorphism between healthy controls and COVID-19-affected patients.

Variant		Genotype Frequency, *n* (%)			*p*-Value	Allele Frequency, n (%)		*p*-Value	HWE
c.20330G>A rs864058		GG	GA	AA	1	G	A	1	
	Controls	89 (98.88)	1 (1.11)	0 (0)		179 (99.44)	1 (0.55)		0.9577
	Patients	86 (98.85)	1 (1.14)	0 (0)		173 (99.42)	1 (0.57)		0.9570

Hardy–Weinberg equilibrium (HWE).

## Data Availability

The data that support the findings of this study are available upon reasonable request from the corresponding author.

## References

[1] Lai C.C., Shih T.P., Ko W.C., Tang H.J., Hsueh P.R. (2020). Severe acute respiratory syndrome coronavirus 2 (SARS-CoV-2) and coronavirus disease-2019 (COVID-19): The epidemic and the challenges. Int. J. Antimicrob. Agents.

[2] Hossain M.G., Javed A., Akter S., Saha S. (2021). SARS-CoV-2 host diversity: An update of natural infections and experimental evidence. J. Microbiol. Immunol. Infect..

[3] Huang C., Wang Y., Li X., Ren L., Zhao J., Hu Y., Zhang L., Fan G., Xu J., Gu X. (2020). Clinical features of patients infected with 2019 novel coronavirus in Wuhan, China. Lancet.

[4] Wang D., Hu B., Hu C., Zhu F., Liu X., Zhang J., Wang B., Xiang H., Cheng Z., Xiong Y. (2020). Clinical Characteristics of 138 Hospitalized Patients with 2019 Novel Coronavirus-Infected Pneumonia in Wuhan, China. JAMA.

[5] Tiecco G., Storti S., Arsuffi S., Antoni M.D., Focà E., Castelli F., Quiros-Roldan E. (2022). Omicron BA.2 Lineage, the “Stealth” Variant: Is It Truly a Silent Epidemic? A Literature Review. Int. J. Mol. Sci..

[6] Hu B., Huang S., Yin L. (2021). The cytokine storm and COVID-19. J. Med. Virol..

[7] Ragab D., Eldin H.S., Taeimah M., Khattab R., Salem R. (2020). The COVID-19 Cytokine Storm; What We Know So Far. Front. Immunol..

[8] Akira S., Uematsu S., Takeuchi O. (2006). Pathogen recognition and innate immunity. Cell.

[9] Kawasaki T., Kawai T. (2014). Toll-Like Receptor Signaling Pathways. Front. Immunol..

[10] Misch E.A., Hawn T.R. (2008). Toll-like receptor polymorphisms and susceptibility to human disease. Clin. Sci..

[11] Dai J., Wang Y., Wang H., Gao Z., Wang Y., Fang M., Shi S., Zhang P., Wang H., Su Y. (2022). Toll-Like Receptor Signaling in Severe Acute Respiratory Syndrome Coronavirus 2-Induced Innate Immune Responses and the Potential Application Value of Toll-Like Receptor Immunomodulators in Patients with Coronavirus Disease 2019. Front. Microbiol..

[12] Zheng M., Karki R., Williams E.P., Yang D., Fitzpatrick E., Vogel P., Jonsson C.B., Kanneganti T.-D. (2021). TLR2 senses the SARS-CoV-2 envelope protein to produce inflammatory cytokines. Nat. Immunol..

[13] Liu Z.-M., Yang M.-H., Yu K., Lian Z.-X., Deng S.-L. (2022). Toll-like receptor (TLRs) agonists and antagonists for COVID-19 treatments. Front. Pharmacol..

[14] Mantovani S., Daga S., Fallerini C., Baldassarri M., Benetti E., Picchiotti N., Fava F., Gallì A., Zibellini S., Bruttini M. (2022). Rare variants in Toll-like receptor 7 results in functional impairment and downregulation of cytokine-mediated signaling in COVID-19 patients. Genes Immun..

[15] van der Made C.I., Netea M.G., van der Veerdonk F.L., Hoischen A. (2022). Clinical implications of host genetic variation and susceptibility to severe or critical COVID-19. Genome Med..

[16] Khanmohammadi S., Rezaei N. (2021). Role of Toll-like receptors in the pathogenesis of COVID-19. J. Med. Virol..

[17] Novelli G., Biancolella M., Mehrian-Shai R., Colona V.L., Brito A.F., Grubaugh N.D., Vasiliou V., Luzzatto L., Reichardt J.K.V. (2021). COVID-19 one year into the pandemic: From genetics and genomics to therapy, vaccination, and policy. Hum. Genom..

[18] Petes C., Odoardi N., Gee K. (2017). The Toll for Trafficking: Toll-Like Receptor 7 Delivery to the Endosome. Front. Immunol..

[19] van der Made C.I., Simons A., Schuurs-Hoeijmakers J., van den Heuvel G., Mantere T., Kersten S., van Deuren R.C., Steehouwer M., van Reijmersdal S.V., Jaeger M. (2020). Presence of Genetic Variants among Young Men with Severe COVID-19. JAMA.

[20] Solanich X., Vargas-Parra G., van der Made C.I., Simons A., Schuurs-Hoeijmakers J., Antolí A., Del Valle J., Rocamora-Blanch G., Setién F., Esteller M. (2021). Genetic Screening for TLR7 Variants in Young and Previously Healthy Men with Severe COVID-19. Front. Immunol..

[21] Peckham H., de Gruijter N.M., Raine C., Radziszewska A., Ciurtin C., Wedderburn L.R., Rosser E.C., Webb K., Deakin C.T. (2020). Male sex identified by global COVID-19 meta-analysis as a risk factor for death and ITU admission. Nat. Commun..

[22] Zaher K., Basingab F., Alrahimi J., Basahel K., Aldahlawi A. (2023). Gender Differences in Response to COVID-19 Infection and Vaccination. Biomedicines.

[23] Peters S.A.E., MacMahon S., Woodward M. (2021). Obesity as a risk factor for COVID-19 mortality in women and men in the UK biobank: Comparisons with influenza/pneumonia and coronary heart disease. Diabetes Obes. Metab..

[24] Yang R., Mei H., Zheng T., Fu Q., Zhang Y., Buka S., Yao X., Tang Z., Zhang X., Qiu L. (2020). Pregnant women with COVID-19 and risk of adverse birth outcomes and maternal-fetal vertical transmission: A population-based cohort study in Wuhan, China. BMC Med..

[25] Tsukahara Y., Hieda Y., Takayanagi S., Macznik A. (2022). Risk Factors for Contracting COVID-19 and Changes in Menstrual and Sleep Cycles in Japanese Female Athletes during the COVID-19 Pandemic. Sports.

[26] Lim S., Sohn M. (2023). How to cope with emerging viral diseases: Lessons from South Korea’s strategy for COVID-19, and collateral damage to cardiometabolic health. Lancet Reg. Health West. Pac..

[27] van der Sluis R.M., Cham L.B., Gris-Oliver A., Gammelgaard K.R., Pedersen J.G., Idorn M., Ahmadov U., Hernandez S.S., Cémalovic E., Godsk S.H. (2022). TLR2 and TLR7 mediate distinct immunopathological and antiviral plasmacytoid dendritic cell responses to SARS-CoV-2 infection. EMBO J..

[28] Dyavar S.R., Singh R., Emani R., Pawar G.P., Chaudhari V.D., Podany A.T., Avedissian S.N., Fletcher C.V., Salunke D.B. (2021). Role of toll-like receptor 7/8 pathways in regulation of interferon response and inflammatory mediators during SARS-CoV2 infection and potential therapeutic options. Biomed. Pharmacother..

[29] Bomba L., Walter K., Soranzo N. (2017). The impact of rare and low-frequency genetic variants in common disease. Genome Biol..

[30] Debnath M., Banerjee M., Berk M. (2020). Genetic gateways to COVID-19 infection: Implications for risk, severity, and outcomes. FASEB J..

[31] Kim Y.C., Jeong B.H. (2020). Ethnic variation in risk genotypes based on single nucleotide polymorphisms (SNPs) of the interferon-inducible transmembrane 3 (IFITM3) gene, a susceptibility factor for pandemic 2009 H1N1 influenza A virus. Immunogenetics.

[32] Kim Y.C., Jeong B.H. (2020). Strong Correlation between the Case Fatality Rate of COVID-19 and the rs6598045 Single Nucleotide Polymorphism (SNP) of the Interferon-Induced Transmembrane Protein 3 (IFITM3) Gene at the Population-Level. Genes.

[33] Kim Y.C., Jeong M.J., Jeong B.H. (2020). Strong association of regulatory single nucleotide polymorphisms (SNPs) of the IFITM3 gene with influenza H1N1 2009 pandemic virus infection. Cell. Mol. Immunol..

[34] Kim Y.C., Jeong M.J., Jeong B.H. (2021). Genetic association between the rs12252 SNP of the interferon-induced transmembrane protein gene and influenza A virus infection in the Korean population. Mol. Cell. Toxicol..

[35] Kim Y.C., Won S.Y., Jeong B.H. (2021). The first association study of single-nucleotide polymorphisms (SNPs) of the IFITM1 gene with influenza H1N1 2009 pandemic virus infection. Mol. Cell. Toxicol..

[36] Huang S., Wei W., Yun Y. (2009). Upregulation of TLR7 and TLR3 gene expression in the lung of respiratory syncytial virus infected mice. Wei Sheng Wu Xue Bao.

[37] Alseoudy M.M., Elgamal M., Abdelghany D.A., Borg A.M., El-Mesery A., Elzeiny D., Hammad M.O. (2022). Prognostic impact of toll-like receptors gene polymorphism on outcome of COVID-19 pneumonia: A case-control study. Clin. Immunol..

[38] El-Hefnawy S.M., Eid H.A., Mostafa R.G., Soliman S.S., Omar T.A., Azmy R.M. (2022). COVID-19 susceptibility, severity, clinical outcome and Toll-like receptor (7) mRNA expression driven by TLR7 gene polymorphism (rs3853839) in middle-aged individuals without previous comorbidities. Gene Rep..

[39] Patel V.K., Paudel K.R., Shukla S.D., Liu G., Oliver B.G., Hansbro P.M., Dua K. (2022). Toll-like receptors, innate immune system, and lung diseases: A vital trilateral association. EXCLI J..

[40] Møller-Larsen S., Nyegaard M., Haagerup A., Vestbo J., Kruse T.A., Børglum A.D. (2008). Association analysis identifies TLR7 and TLR8 as novel risk genes in asthma and related disorders. Thorax.

[41] Roponen M., Yerkovich S.T., Hollams E., Sly P.D., Holt P.G., Upham J.W. (2010). Toll-like receptor 7 function is reduced in adolescents with asthma. Eur. Respir. J..

[42] Zhang Q., Qian F., Zhou L., Wei G., Wang Y., Hu Z., Jin G., Bai J., Yin K. (2009). Polymorphisms of TLR7 and TLR8 associated with risk of asthma and asthma-related phenotypes in a southeastern Chinese Han population. J. Nanjing Med. Univ..

[43] Nilsson D., Andiappan A.K., Halldén C., De Yun W., Säll T., Tim C.F., Cardell L.-O. (2012). Toll-like receptor gene polymorphisms are associated with allergic rhinitis: A case control study. BMC Med. Genet..

[44] Kennedy R.B., Ovsyannikova I.G., Haralambieva I.H., O’Byrne M.M., Jacobson R.M., Pankratz V.S., Poland G.A. (2012). Multigenic control of measles vaccine immunity mediated by polymorphisms in measles receptor, innate pathway, and cytokine genes. Vaccine.

[45] Stark J.R., Wiklund F., Grönberg H., Schumacher F., Sinnott J.A., Stampfer M.J., Mucci L.A., Kraft P. (2009). Toll-like receptor signaling pathway variants and prostate cancer mortality. Cancer Epidemiol. Biomark. Prev..

[46] Alhabibi A.M., Hassan A.S., Elbaky N.M.A., Eid H.A., Khalifa M., Wahab M.A., Althoqapy A.A., Abdou A.E., Zakaria D.M., Nassef E.M. (2023). Impact of Toll-Like Receptor 2 and 9 Gene Polymorphisms on COVID-19: Susceptibility, Severity, and Thrombosis. J. Inflamm. Res..

[47] Alaa A., Sarhan N., El-Ansary M.G.L., Bazan N.S., Farouk K., Ismail R.S., Schalaan M.F., Abd-Allah A.R.A. (2023). Association between genetic polymorphism, severity, and treatment response among COVID-19 infected Egyptian patients. Front. Pharmacol..

[48] Salamaikina S., Karnaushkina M., Korchagin V., Litvinova M., Mironov K., Akimkin V. (2022). TLRs Gene Polymorphisms Associated with Pneumonia before and during COVID-19 Pandemic. Diagnostics.

